# Waterpipe use among the youth in Ghana: Lessons from the Global Youth Tobacco Survey (GYTS) 2017

**DOI:** 10.18332/tid/120937

**Published:** 2020-05-29

**Authors:** Divine D. Logo, Sardick Kyei-Faried, Felix B. Oppong, Kenneth A. Ae-Ngibise, Joana Ansong, Seidu Amenyaglo, Sampson T. Ankrah, Arti Singh, Ellis Owusu-Dabo

**Affiliations:** 1 Research and Development Division, Ghana Health Service, Accra, Ghana; 2 Department of Global and International Health, School of Public Health, College of Health Sciences, Kwame Nkrumah University of Science and Technology, Kumasi, Ghana; 3 Disease Control and Prevention Division, Ghana Health Service, Accra, Ghana; 4 Kintampo Health Research Centre, Ghana Health Service, Kintampo, Ghana; 5 School of Medicine and Public Health, Faculty of Health and Medicine, University of Newcastle, Newcastle, Australia; 6 World Health Organization Ghana Country Office, Accra, Ghana

**Keywords:** tobacco, waterpipe, youth, junior high school, Ghana

## Abstract

**INTRODUCTION:**

The Global Youth Tobacco Survey’s findings have been used to support Ghana’s tobacco control legislation, monitor tobacco use among the youth and also used in meeting various Articles of the World Health Organization (WHO) Framework Convention on Tobacco Control (FCTC). These Articles include: Article 8 (Protection for exposure to tobacco smoke); Article 12 (Education, communication, training and public awareness); Article 13 (Tobacco advertising, promotion, and sponsorship); Article 14 (Demand reduction measures concerning tobacco dependence and cessation); and Article 16 (Sales to and by minors). Among the four waves of GYTS in Ghana, the 2017 GYTS was the first to assess waterpipe smoking, through optional questions included in the GYTS questionnaire. We assessed sex, age and regional differentials in waterpipe smoking among the youth in Ghana, and also explored the association between the use of other tobacco products and waterpipe use.

**METHODS:**

The GYTS employs a standardized methodology with self-administered questionnaires, consisting of core, optional, and country specific questions. Fourteen questions, out of the seventy-four (74) questions administered for the entire GYTS, assessed waterpipe tobacco smoking (WTS). Chi-squared test was used to assess sex, age, grade/form and regional differentials in waterpipe use. Furthermore, the association between smoking cigarettes, smokeless tobacco, electronic cigarettes and waterpipe smoking, was explored by employing a chi-squared test with a 5% significance level.

**RESULTS:**

Of a total of 5664 students who participated in the study, 90.9% were aged 13–15 years. The respondents were almost equally distributed among males and females. Overall, 3.1% of the respondents had ever smoked waterpipe. The overall prevalence of current waterpipe use was 1.7%; with 2.1% in girls (95% CI: 0.9–4.7%) and 0.9% in boys (95% CI: 0.5–1.6%), p=0.033. Additionally, more than half (55.0%) of the current waterpipe users smoked three or more sessions per day. Surprisingly, close to half (46.9%) of the current waterpipe users smoked at home.

**CONCLUSIONS:**

Waterpipe use, particularly among the female student population, represents an emerging tobacco epidemic and hence deserves immediate attention from authorities. This study revealed that waterpipe is being used among Junior High students in Ghana. Education on the health implications of waterpipe use should be intensified among the youth, to help minimize its use and to prevent its associated health harms.

## INTRODUCTION

Tobacco use remains a huge public health concern. It kills more than 8 million people every year, of which, more than 7 million are as a result of direct tobacco use and close to 1.2 million due to exposure of non-smokers to secondhand smoke^1^. Africa is considered to be at the beginning of the tobacco-use epidemic, however, there is a significant increase in the prevalence of tobacco use, especially among the youth. This is the focus of tobacco industry, to recruit new smokers by taking advantage of a government unable to implement appropriate tobacco control measures^2^.

Even though cigarette smoking is considered the main form of tobacco use in most parts of the world, waterpipe tobacco smoking (WTS) is becoming extensively used worldwide, with high prevalence in the Middle East, Asia and Africa. It is worth noting that waterpipe use has exceeded cigarette smoking among men and women, and is very common among the youth, in some countries in the Eastern Mediterranean Region (EMR)^3^.

Tobacco is manufactured and used in various forms including cigarettes, cigars, chewable tobacco, snuff, bidis, and waterpipe smoking, also known as hookah, shisha, goza, nargile^4^ in different cultures and countries. Waterpipe was invented in ancient India purportedly as a safer means of smoking tobacco, with the assumption that the water filtering system may filter out the harmful chemicals^5^. Contrary to the beliefs that WTS is less harmful and less addictive compared to cigarettes, studies have shown that waterpipe smoke contains many toxicants as in cigarette smoke and it is also harmful, addictive, serves as a gateway to cigarettes, as well as ruins cessation efforts^6^.

In a study conducted among the youth in school in Tunisia (aged 13–17 years), the prevalence of ever and current smoking of waterpipe was reported to be 19.3% and 5.2%, respectively^7^. Also, a similar study that analysed the GYTS data on waterpipe use from 16 Arab countries showed that overall, one in 10 Arab youth aged 13–15 years currently smokes waterpipe, with higher prevalence among boys^8^.

Even though there is detailed information regarding waterpipe smoking among the youth in both developed countries and some LMICs, there is very little information on how common this form of tobacco is used among Ghanaian youth, and their level of knowledge about its health harms.

Although limited data exist, there are anecdotes from media sources and some unpublished data suggesting increasing waterpipe use. For example, it was reported that there are 7.14% first time waterpipe smokers and 36.7% current smokers of both cigarette and waterpipe in some communities in the capital of Ghana. That study had respondents whose mean age was 26.7 ± 5.3 and a sample size of 210 people^9^.

Until the advent of the Global Tobacco Surveillance System (GTSS), where the Global Youth Tobacco Survey (GYTS) helped to bridge the information gap, especially in LMICs^10^, Ghana had no nationally representative data on tobacco use. Ghana, therefore, benefited from four waves of the GYTS in the years 2000, 2006, 2009 and 2017, respectively^11^. The GYTS findings together with the Ghana demographic surveys^12,13^ assisted Ghana in meeting its obligations as a party to the WHO FCTC^14^ and also supported the passage of national law for tobacco control (Public Health Act, 2012; LI 2247, 2017). The findings from previous GYTS also assisted Ghana to be in compliance with the WHO FCTC Articles such as: Article 8 (Protection for exposure to tobacco smoke); Article 12 (Education, communication, training and public awareness); Article 13 (Tobacco advertising, promotion, and sponsorship); Article 14 (Demand reduction measures concerning tobacco dependence and cessation); and Article 16 (Sales to and by minors)^14^, just to mention few. Apart from these, Ghana also ratified the WHO FCTC as part of the Public Health Act and fully enforced and strengthened by the recent passage of the tobacco control legislative instrument L.I. 2247.

This paper is a secondary analysis of the 2017 Ghana-GYTS and aimed at the following. First, to assess the prevalence of waterpipe use and also report on the sex, age and regional differentials in waterpipe smoking among the youth in Ghana. Second, to explore the association between the use of other tobacco products, namely cigarettes, smokeless tobacco, electronic cigarettes and waterpipe.

## METHODS

### Study population

The data used in this study are from the 2017 Ghana Global Youth Tobacco Survey (Ghana GYTS), which was implemented by the Ministry of Health (MOH), Ghana Education Service (GES) and the World Health Organization (WHO). The survey was carried out among a representative sample of Junior High students in Ghana. Students were sampled from the three main ecological/epidemiological zones of Ghana, namely the savanna or the northern zone, the middle or the forest zone, and the coastal zone. The savannah/northern zone covers the current five northern regions (Northern, Savannah, North East, Upper East and Upper West regions). The middle/forest zone comprises the current Ashanti, Bono, Bono East, Ahafo, Eastern, Volta and Oti regions, and the coastal zone covers the current Greater Accra, Central, Western, and Western North regions.

Sampling frame included all Junior High schools, both public and private, in Ghana. A list of all Junior High Schools (JHS) in Ghana was obtained from Ministry of Education/Ghana Education Service for sampling. The selection of study participants was done in two stages, first within each ecological zone, and then the schools randomly selected using probability proportional to enrollment size. The least enrollment size was tagged at 80 students. The schools were the primary sampling unit for the study. A seventy-seven (77) cluster of schools was chosen, comprising 26 for the northern/savanna, 25 for the forest/middle, and 26 for the coastal belts. The total number of students who participated was 5664. Parental consent and assent from students were sought before data collection and participation. Students were also informed that participation was voluntary and therefore they could discontinue when they wished, or decide not to respond to a question that he or she was not comfortable with.

### Data collection

Students participated in the study on a voluntary basis and data were collected anonymously using a pre-validated self-administered questionnaire. The GYTS questionnaire included in-country adaptation of contextual country specific questions, as applicable. The survey tool assessed knowledge and attitudes of young people towards cigarette smoking, proportion of cigarette smoking and other tobacco products use among the youth, role of the media and advertising in young people’s use of cigarettes, access to cigarettes, tobacco-related school curriculum, secondhand smoke, cessation of cigarette smoking, electronic cigarette use, waterpipe use, and awareness of the Ghana Public Health Act 851. Data on the demographic characteristics of the respondents were also collected. The data collection was supervised by trained research assistants and the class teachers of the selected schools. Ethical approval for the study was obtained from the Ghana Health Service Ethics Review Committee (GHS-ERC: 05/03/17). Approval was also obtained from the Ministry of Education/Ghana Education Service and the heads of the selected schools. We obtained parental consent and written assent from children after a detailed explanation of the study objectives, procedures, risks and benefits had been presented to them.

### Questionnaire for waterpipe

This is the first time Ghana had ever assessed waterpipe use among the youth in the GYTS. Centers for Disease Control (CDC) provided optional questions for countries undertaking the GYTS, based on their country needs for tobacco control. Among the options were electronic cigarette, waterpipe, bidi and smokeless tobacco modules, out of which Ghana decided to assess waterpipe and electronic cigarette use among the youth. All the optional questions were pre-designed and were not to be adapted, but rather to be included in full. There were fourteen questions in the waterpipe module. The following were sampled: Question 1 (Have you ever tried or experimented with waterpipe smoking, even one or two puffs?) to which respondents who answered ‘yes’ were assessed for ever smoked waterpipe. While Question 3 (During the past 30 days, on how many days did you smoke waterpipe?) to which those who responded to one were defined as current smokers.

### Statistical analysis

Data were weighted by taking into account the design characteristics of the survey. While we administered the questions to youth with ages ranging from 11–17 years, we present results nationally representative of those students aged 13–15 years, consistent with GYTS worldwide. The background characteristics of the study participants were presented as unweighted frequencies, weighted frequencies, and weighted percentages. The prevalence of waterpipe use was stratified by background characteristics of the respondents. Chi-squared test was used to assess the association between participant’s characteristics namely sex, age, grade/form and region and ever using waterpipe. Similarly, the association between these background characteristics and current waterpipe use was assessed using chi-squared test. Current use of any of the tobacco products implies usage anytime during the past 30 days, whereas ever use implies ever smoking any of the tobacco products mentioned, even one or two puffs. The relationship between smoking cigarette, smokeless tobacco, electronic tobacco and waterpipe use as well as the association between exposure to secondhand smoke and waterpipe use were also explored using a chi-squared test. The test results were considered to be statistically significant at a default alpha of 5%. STATA version 14 (STATA Corp, Texas) was used for the statistical analysis.

## RESULTS

The overall response rate was 93.8%, and a total of 5664 eligible students in Junior High School 1–3 completed the survey, of which 137, 5116 and 402 were aged 11–12 years, 13–15 years and 16–17 years, respectively. Results are reported for all the participants of the survey with focus on the 13–15 years age group. Table 1 presents the weighted and unweighted number of participants as well as the weighted percentage distribution based on participant’s background characteristics. With respect to sex, there was almost equal representation of both males and females. Majority of the respondents were within the 13–15 years age group (90.9%). Most of the respondents were from the middle/forest zone (47.5%).

**Table 1:** Background characteristics of study participants, GYTS 2017, Ghana

*Characteristics*	*Unweighted number*	*Weighted number*	*Weighted percentage*
**Sex^a^**			
Male	2707	2878	51.1
Female	2929	2758	48.9
**Age (years)^b^**			
11–12	137	153	2.7
13–15	5116	5139	90.9
16–17	402	363	6.4
**Grade^c^**			
First	2104	2115	37.5
Second	2873	1921	34.1
Third	662	1603	28.4
**Region**			
Savanna/northern zone	1862	993	17.5
Middle/forest zone	1804	2691	47.5
Coastal zone	1998	1980	35.0

a 28 missing data.

b 25 missing data.

c 9 missing data.

### Waterpipe use by background characteristics

Table 2 presents the prevalence of waterpipe use by background characteristics of respondents. A total of 5052 students responded to the questions on ever using waterpipe and 5370 on current use of waterpipe. There was no sex difference in the proportion of respondents who have ever used waterpipe. However, among the three age groups, there was a significant difference in the proportion of respondents who have ever used waterpipe. Likewise, there was a statistically significant difference in waterpipe use among respondents from the three regions, with more users in the savanna/northern zone compared to the other regions. Among the current users of waterpipe, differences were observed for all the background characteristics. Particularly, more females (2.1%) than males (0.9%) were current smokers of waterpipe; more of the 11–12 years old adolescents smoked waterpipe compared to those in the older age groups, current waterpipe use was higher among third grade students and was also higher among respondents from the savanna/northern zone.

**Table 2:** Prevalence of waterpipe use by background characteristics of respondents, GYTS 2017, Ghana

*Characteristics*	*Ever users*			*Current users*		

	*n/N ^b^*	*% (95% CI) ^c^*	*p ^a^*	*n/N ^b^*	*% (95% CI) ^c^*	*p ^a^*
**Overall**	143/5052	3.1 (2.0–4.8)		77/5370	1.7 (0.9–3.3)	
**Sex**						
Male	72/2402	3.1 (2.1–4.5)	0.677	31/2565	0.9 (0.5–1.6)	0.033
Female	68/2627	2.6 (1.2–5.5)		44/2779	2.1 (0.9–4.7)	
**Age** (years)						
11–12	9/105	9.6 (3.5–23.7)	0.033	10/120	15.7 (6.2–34.7)	<0.001
13–15	117/4609	2.8 (1.7–4.4)		56/4879	1.3 (0.6–2.8)	
16–17	17/332	5.9 (2.2–14.9)		11/364	2.1 (1.0–4.3)	
**Grade**						
First	67/1859	2.2 (1.4–3.5)	0.025	33/1954	0.8 (0.5–1.4)	<0.001
Second	63/2577	2.3 (1.5–3.3)		32/2750	0.8 (0.5–1.2)	
Third	12/600	5.4 (2.5–11.2)		12/647	4.0 (1.5–10.1)	
**Region**						
Savanna/northern zone	90/1525	11.3 (6.2–19.7)	<0.001	62/1645	7.5 (3.4–16.1)	<0.001
Middle/forest zone	31/1658	2.0 (1.0–4.0)		7/1764	0.7 (0.2–3.3)	
Coastal zone	22/1869	1.2 (0.6–2.4)		8/1961	0.5 (0.2–1.2)	

a p-value for difference in proportion based on background characteristics.

b Absolute numbers.

c Weighted.

### Age, place and sessions per day of first waterpipe usage among participants

Table 3 presents the results on age at first use of waterpipe, number of days waterpipe was smoked by the current waterpipe users, number of sessions of waterpipe smoking in a day, and where waterpipe smoking activities usually took place. A significant majority of the respondents (67.3%) first used waterpipe when they were 13 years old or younger. Also, among both males and females, most of the respondents first used waterpipe when they were 13 years old or younger. With respect to number of days students used waterpipe in the past month preceding the survey, most of the current smokers reported using waterpipe at least a day or 2 in a month. Also, 13.8% of the current smokers reported smoking waterpipe everyday (30 days). For waterpipe smoking sessions in a day, most of the current smokers (40.5%) reported having 3 sessions per day, while about a quarter reported one session per day. Among the male users, most of them smoked four or more sessions per day. Majority of the respondents smoked waterpipe in the home environment (46.9%).

**Table 3:** Waterpipe use: place of smoking, smoking in the past 30 days, and smoking sessions per day, GYTS 2017, Ghana

*Variables*	*Total n (%)*	*Male n (%)*	*Female n (%)*
**Age at first waterpipe use^a^ (years)**			
≤13	54 (67.3)	17 (52.1)	35 (77.2)
≥14	26 (32.7)	16 (47.9)	10 (22.8)
Total	80 (100)	33 (100)	45 (100)
**Number of days waterpipe was smoked in past month^b^**			
1–2	32 (41.4)	6 (19.4)	17 (37.6)
3–5	14 (17.9)	9 (27.6)	7 (17.1)
6–9	6 (8.1)	6 (20.5)	2 (4.6)
10–19	1 (2.0)	1 (3.1)	1 (2.0)
20–29	13 (16.8)	7 (22.4)	8 (18.5)
30	11 (13.8)	2 (7.0)	9 (20.2)
Total	77 (100)	31 (100)	44 (100)
**Waterpipe smoking sessions per day^b^**			
1	13 (24.5)	6 (25.7)	7 (23.0)
2	11 (20.5)	9 (36.8)	3 (12.2)
3	22 (40.5)	1 (2.2)	18 (61.1)
≥4	8 (14.5)	8 (35.3)	1 (3.7)
Total	54 (100)	24 (100)	29 (100)
**Where waterpipe was smoked^b^**			
Home	25 (46.9)	5 (30.0)	12 (35.9)
Coffee shop	10 (20.0)	2 (13.6)	10 (28.2)
Restaurant	5 (9.6)	4 (19.0)	3 (9.6)
Bar or club	7 (14.1)	-	8 (22.9)
Other	5 (9.4)	7 (37.4)	1 (3.5)
Total	53 (100)	18 (100)	34 (100)

a Based on those who have ever smoked waterpipe.

b Based on current waterpipe smokers.

### Tobacco-use profile of respondents

Tobacco use profile, namely current cigarette smoking, smokeless tobacco and electronic cigarette smoking, is presented in Table 4. The prevalence of current cigarette smoking, smokeless tobacco use and electronic cigarette smoking was 3.0%, 3.6% and 5.8%, respectively. Also, 9.9% were at the time of the survey smoking either cigarettes, smokeless tobacco, electronic cigarettes or waterpipe. Details of the tobacco-use profile by background characteristics are presented in Table 4.

**Table 4:** Current tobacco-use profile of respondents, GYTS 2017, Ghana

*Characteristics*	*Cigarette*		*Smokeless tobacco*		*Electronic cigarette*		*Any tobacco ^c^*	

	*n/N ^a^*	*% ^b^*	*n/N ^a^*	*% ^b^*	*n/N ^a^*	*% ^b^*	*n/N ^a^*	*% ^b^*
**Overall**	162/5215	3.0	211/5357	3.6	349/5430	5.8	582/ 5659	9.9
**Sex**								
Male	92/ 2475	3.2	104/2555	3.2	148/2588	5.5	288/2706	9.9
Female	66/ 2651	2.7	105/2780	4.0	198/2816	6.1	287/2926	9.4
**Age** (years)								
11–12	9/116	10.5	8/122	8.5	23/127	21.6	32/136	28.1
13–15	136/4736	2.8	172/4850	3.1	270/4903	4.9	472/5112	9.0
16–17	17/354	2.7	30/376	8.0	56/391	11.0	77/402	15.4
**Grade**								
First	80/1908	3.3	78/1970	2.6	166/1989	4.7	263/2102	8.5
Second	66/2668	2.4	111/2729	3.6	155/2782	4.4	264/2872	8.1
Third	11/619	3.2	20/638	4.7	21/637	8.8	47/661	14.0
**Region**								
Savanna/northern zone	88/1602	7.6	125/1703	9.1	261/1736	22.6	368/1858	28.3
Middle/forest zone	43/1702	2.5	50/1720	3.0	48/1747	2.9	119/1803	7.0
Coastal zone	31/1911	3.0	36/1934	1.8	40/1947	1.9	95/1998	4.8

a Absolute numbers.

b Weighted percentage.

c Includes current use of cigarettes, smokeless tobacco, electronic tobacco and/or waterpipe.

### Waterpipe and other tobacco use

The use of other tobacco products was significantly associated with current waterpipe use (Table 5). Among the current cigarette smokers, 18.2% were also current waterpipe smokers, while 0.6% of the non-cigarette smokers smoked waterpipe anytime during the past 30 days prior to the survey. Likewise, more current smokeless tobacco users as well as current electronic cigarette smokers were current waterpipe smokers compared to those who were not current smokers of smokeless tobacco or electronic cigarettes. Similar results were obtained among males and females. On exposure to secondhand smoke at home, 6.0% of those exposed used waterpipe, while 0.5% of the students who were not exposed to cigarette smoke at home also used waterpipe. Similar results were obtained among both males and females.

**Table 5:** Association between use of other tobacco products and waterpipe smoking, GYTS 2017, Ghana

*Characteristics*	*All waterpipe users (N=77)*			*Male waterpipe users (N=31)*			*Female waterpipe users (N=44)*		

	*n/N*	*% (95% CI)*	*p*	*n/N*	*% (95% CI)*	*p*	*n/N*	*% (95% CI)*	*p*
**Current cigarette smokers**									
Yes	22/111	18.2 (8.7–34.2)	<0.001	11/75	8.9 (4.4–17.3)	<0.001	11/32	33 (15.1–58.8)	<0.001
No	17/4927	0.6 (0.2–1.8)		4/2319	0.2 (0.1–1.1)		12/2606	0.5 (0.2–1.9)	
**Current smokeless tobacco users**									
Yes	23/163	9.4 (4.7–17.9)	<0.001	7/86	9.8 (3.4–25.3)	<0.001	16/75	9.4 (4.8–17.4)	<0.001
No	34/4949	1.0 (0.4–2.5)		16/2352	0.3 (0.2–0.6)		17/2578	1.2 (0.3–4.1)	
**Current electronic cigarette**									
Yes	51/187	32.9 (18.3–51.9)	<0.001	19/75	16.8 (8.5–30.5)	<0.001	31/109	47 (25.3–70.2)	<0.001
No	11/4992	0.5 (0.2–1.6)		4/2389	0.2 (0.1–1.0)		6/2581	0.3 (0.1–1.1)	
**Exposure to secondhand smoke at home**									
Yes	58/1052	6.0 (2.8–12.3)	<0.001	21/521	3.1 (1.4–6.4)	<0.001	36/519	9.7 (4.1–21.5)	<0.001
No	19/4308	0.5 (0.2–1.7)		10/2039	0.2 (0.1–0.5)		8/2255	0.2 (0.1–0.4)	

### Waterpipe use cessation

Of the current waterpipe users, 55% reported having been refused waterpipe by vendors in the past 30 days prior to the survey because of their age. Also, 72% of the current waterpipe users reported that they wanted to quit smoking waterpipe. However, 68.3% of the current waterpipe users had ever tried to stop smoking waterpipe during the past 12 months prior to the survey.

## DISCUSSION

This paper used data from the 2017 Global Youth Tobacco Survey (GYTS), to provide detailed results on waterpipe smoking in a nationally representative sample of adolescents in Junior High school in Ghana. Close to 1 in 10 students currently uses any form of tobacco products. A little less than 3 in 100 students currently smoke cigarettes and smokeless tobacco, and about 5% use electronic cigarettes. Waterpipe smoking however, was the lowest among students in Ghana relative to other tobacco use^11^. This low prevalence of WTS was inconsistent with many studies including a systematic review on the GYTS results in the Eastern Mediterranean Region, among 17 countries^15-17^. This finding could be due to the increasing trend of waterpipe smoking among the youth globally, including females, and largely as a result of misconceptions about the harms^16,18^. Within Sub-Saharan Africa (SSA), higher waterpipe use prevalence of 8.1% among adolescents in school was reported in Gambia, where relatively high prevalence was also observed among girls (11.4% of boys and 5.4% of girls)^19^. This also supports the finding that tobacco use among Ghanaians is lower compared to other SSA and high-income countries^20^.

Even though Ghana reported low WTS^21^, the current prevalence is an indication of acceptance of the practice among the youth, especially the females, even though the practice is not recognized as part of the Ghanaian culture as is the case in the Eastern Mediterranean Region and North African countries.

These findings provide confirmation of juvenile waterpipe smoking, globally. In Ghana, even though WTS is relatively low compared to other countries, especially in the EMR, it is of public health concern and an indication that waterpipe outlets do not prevent sales to those who are underage. The Public Health Act 851 of Ghana prevents access to any form of tobacco products for any minor below the age of 18 years. The findings show the weaknesses in the enforcement of the Act. Authorities must use these findings to address the problem of teen waterpipe access and smoking in the country. Retailers should play a key role in preventing adolescents from getting access to WTS. Furthermore, tobacco control efforts must target both sexes, but must however focus on girls.

Regarding gender and age of initiation of WTS, our study shows more girls were indulging in WTS and a high proportion started at a very young age. This finding is consistent with the work of Alzyoud et al.^22^ in Jordan, but with a higher prevalence compared to Ghana. In the EMR^17^ however, it has been reported that there was equal prevalence among boys and girls, while many studies^15-16^ reported waterpipe to be gender-specific, with girls likely to smoke waterpipes more than boys. The issue of girls leading in WTS must be highly considered by all stakeholders since in many African social cultures, and across the globe, smoking is generally more acceptable among men than in women^17,19^. Gender and age of initiation of WTS was mainly among those aged 13 years and below and also higher among girls. Similar findings were reported in Saudi Arabia where 68.8% of students start smoking waterpipe at the ages of 16–18 years, with more male smokers than females^23^. However, in Jordan, the situation was similar to our study where more females were reported smoking waterpipe than males^22^.

In our study, about one in three of the waterpipe users smoked for 10 or more days out of 30 days prior to the survey. Also, more than 70% of the respondents smoked 2 or more sessions per day. Studies have revealed that one session of waterpipe smoking contains approximately 200 puffs of smoke, which exposes smokers to 3 to 6 times higher levels of carbon monoxide and 46 times higher levels of tar^24,25^. In a systematic review on the effects of WTS on health outcomes, it was revealed that WTS was significantly associated with lung cancer, respiratory illness, lowbirthweight and periodontal diseases^26^. These health outcomes were supported by a similar review and subsequently associated WTS further to bronchitis and wheezing, due to exposure to secondhand WTS, oral cancer, metabolic syndrome, cardiovascular diseases and mental health^27^. Given these adverse outcomes associated with WTS, the government, authorities, teachers, parents and guardians should all pay close attention to the welfare of adolescents and educate them on the consequences of WTS.

We further found that the respondents’ homes were the most common place where waterpipe was recently smoked. Waterpipe gadgets are not portable devices, hence are very difficult to conceal compared with other tobacco products. The youth smoking at home suggests the practice might involve parents, relatives or caregivers who might also be waterpipe smokers who support their children to smoke waterpipe at home and even at social gatherings^16^.

Considering the regional disparities in WTS, the savanna/northern zone reported the highest use. This is consistent with the country’s Demographic and Health Survey findings^12,13^ as being the region with the highest tobacco use. In Ghana, the savanna/northern zone is the most deprived and poorest region where most of the communities are rural settlements. This finding agrees with the results of a study conducted in Vietnam where rural residence was associated with waterpipe smoking^28^.

Our study also found that cigarette smoking and smokeless tobacco use were associated with waterpipe smoking. These findings agree with synergism in the indulgence of risky behaviour such as waterpipe smoking. These findings also agree with the results of other studies that have explored the association between waterpipe and cigarette smoking^29,30^. In this study, electronic cigarette smoking was also found to be associated with waterpipe smoking. This result is supported by a study conducted among the youth in Australia where most students (65%) using e-cigarettes and waterpipe-tobacco (67%) had also used tobacco cigarettes^31^.

We also found a significant association between waterpipe smoking and exposure of students to secondhand smoke at home. Among adolescents unexposed to secondhand smoke at home, 0.5% were waterpipe smokers. On the other hand, 6.0% of those exposed to secondhand smoke at home smoked waterpipe anytime during the past 30 days prior to the survey. The misconceptions about the harmful effects associated with WTS could be the passageway leading to the high propagation of the practice, especially among the youth. In a related study supporting this finding, youth with a low risk of smoking increased susceptibility while experimenting with alternative tobacco, hence predisposing them to future tobacco use^32^.

We also found that more than three-quarters of waterpipe smokers attempted quitting WTS. Nicotine addiction is reported to be associated with the length of time one has been smoking, and difficulty in quitting^33^. This suggests a determined effort by the Ministry of Health and all relevant stakeholders to support waterpipe smokers with effective cessation programmes targeting both sexes with a focus on girls. These programmes could be in the form of awareness creation and education about the harmful effects of WTS among the youth in the schools and also incorporated into school health education curricula. School-based smoking prevention programmes are reported to be the most effective strategies to reduce smoking among adolescents, and have shown to reduce smoking intention and behaviour^34^. Also, there should be a strict regulation of waterpipe outlets to prohibit selling to minors as enshrined in the Ghana Public Health Act 851. Waterpipe must also be regulated, just as cigarette products are, to limit access by minors. This will prevent the youth turning to waterpipe as an alternative to cigarette smoking.

Our study also revealed that more than half of the current waterpipe smokers were ever refused sale of waterpipe due to their age. It has been reported that adolescents who are refused sale of tobacco due to their age have a significantly higher chance of quitting smoking^35^. This implies that many of the youth smoking waterpipe may quit if only adequate education on awareness and support for cessation are available and provided. These findings also suggest that the Ministry of Health/Ghana Health Service Health Promotion Division should include a comprehensive tobacco awareness component in their programmes that will include WTS and other nicotine delivery devices such as electronic cigarettes targeting the youth both in and out of school.

### Strengths and limitations

Our study has a number of strengths. The key among them is the large sample size, which was nationally representative and with high response rates among schools and students. The participation rate among those sampled was extremely high and the sample is highly representative of the total population in this age group. Additionally, all the invited schools participated in this study. However, limitations associated with the use of self-administered questionnaires by students may have led to under/over reporting. Yet some studies have reported high reliability of the results on self-administered youth smoking questionnaires^36,37^. Also, the survey was limited to youth in school only. This however may not represent all youth in Ghana aged 13–15 years. Finally, the findings from this study indicate the need to assess patterns of waterpipe smoking among the youth in Ghana, especially in secondary and tertiary education.

## CONCLUSIONS

Even though WTS among the youth is considerably low compared to other countries, it is important for Ghana to be concerned and prepared to avert high prevalence since the practice is gradually becoming a global public health challenge. There is, therefore, the need to not only design programme interventions to ‘nip this canker in the bud’ but also implement strict enforcement of the public health law on smoking in public places.
